# Editorial

**Published:** 1871-01

**Authors:** 


### Editorial.

American Medical Association.—All who have faithfully attended the meetings of the Association, and participated in its doings with a sincere desire to make it the instrument of advancing the scientific, as well as social interests of the whole profession, have felt the embarrassments consequent on the incomplete preparation of many papers and reports on the part of those who present them; the absence of all knowledge at the opening of each annual meeting, in reference to the special reports and papers that are to be presented in each section, and the order in which they are to come up for consideration. The Association meets in general session the first morning, and after the preliminary business is disposed of, the list of special committees is called, and opportunity for the announcement of volunteer papers given, and such reports and papers as are announced by their titles are referred to the appropriate sections. By the time this work is completed, it is, perhaps, within an hour of the time for the sections to meet. Unless the authors of reports and papers are watching closely the action of the Association, and taking particular pains to learn early the location ef the several section-rooms, they often loose a part or all of the first session of the section before they find out where they are expected to present the results of their labor; and the great majority of the members are still more in the dark as to the time when, and place where, any particular report or paper is to be read and discussed. Efforts have been made, and rules adopted from time to time, for the express purpose of removing these embarrassments.

And if the present by-laws of the Association were generally understood and adhered to, they would be entirely obviated and the value of the transactions greatly increased. We would call special attention to a by-law that was adopted and first became operative at the last annual meeting. After providing for the more perfect and efficient organization of the sections by the election of their officers at the same time, and in the same manner as the officers of the general body, it further provides as follows:

"Papers appropriate to the several sections, in order to secure consideration and action, must be sent to the *Secretary* of the apprcpriate *Section*, at least *one month* before the meeting which is to act upon them. It shall be the duty of the Secretary, to whom such papers are sent, to examine them with care, and, with the advice of the Chairman of his section, to determine the time and order of their presentation, and give due notice of the same; and, after their full examination and discussion by the section, they shall be sent to the Permanent-Secretary of the Association."

If writers, in compliance with this rule, would thus place their papers in the hands of the Secretary of the section in which they wish to have them read and considered, three or four weeks in advance of the annual meeting, it would enable each Secretary to place on a bulletin-board in the public hall, at the opening of each annual meeting, the titles of the several papers and the order of their reading in each section. This would secure a prompt and fair attendance in each section-room and a fair consideration of each paper or report. To facilitate this work, we append the names and residences of the present officers of the several sections, as follows:

*Chemistry and Materia Medica*—Chairman, Dr. D. W. Yandell, of Louisville, Ky.; Secretary, Dr. H. S. Hurd, of Galesburg, Ill.

*Practical Medicine and Obstetrics*—Chairman, Dr. H. R. Storer, of Boston, Mass.; Secretary, Dr. J. K. Bartlett, of Milwaukee, Wis.

*Surgery and Anatomy*—Chairman, Dr. J. L. Atlee, of Lancaster, Pa.; Sectretary, M. Horace Carpenter, of Salem, Oregon.

*Meteorology, Medical Topography, and Epidemics*—Chairman, Dr. N. S. Davis, of Chicago, Ill.; Secretary, Dr. C. C. Hildreth, of Zanesville, Ohio.

*Medical Jurisprudence, Physiology, and Hygiene*—Chairman, Dr. Theophalus Parvin, of Indianapolis, Ind.; Secretary, Dr. J. A. Murphy, of Cincinnati, Ohio.

*Psychology*—Chairman, Dr. J. H. Griscom, of New York City; Secretary, Dr. O. F. Rennick, of Mo.

If our brethren of the medical press, in all parts of the country, would now call the attention of their readers to the necessity of more rigidly complying with the by-laws to which we have alluded, they would do more good than by any amount of complaining *after* the next meeting.

---

OPHTHALMOLOGY AND OTOLOGY.—Samuel J. Jones, M.D., Professor of Ophthalmology and Otology in the Chicago Medical College, has been appointed one of the surgeons to Mercy Hospital, and has charge of all cases of diseases involving the eye and ear.

We often receive letters asking for information in regard to the best place for patients to be well cared for, and at the same time secure special treatment for the organs just named.

To all such inquiries we would say, that we know of no better place in our country than the wards of the Mercy Hospital in this city.

THE VOLUME FOR 1870–71.—We have a few complete copies of Volume 11 of the *Examiner*, for 1870. And to any new subscriber, who will send us *five* dollars, we will send, postage paid, the volume for 1870 at once, and the monthly numbers for 1871 as they are issued. The present number commences the *twelfth* volume, and affords a good opportunity for new subscribers to commence.

### Mortality for the Month of November, 1870.

| | | | | | |
|---|---|---|---|---|---|
| Accidents, Asphyxia | 3 | Diarrhœa | 2 | " abscess of | 1 |
| " burns | 1 | " chronic | 4 | " inflammation of | 1 |
| " drowned | 1 | Delirium Tremens | 2 | " hypertrophy of | 2 |
| " fracture of skull | 1 | Diphtheria | 19 | Lungs, congestion | 1 |
| " thrown from wagon | 1 | Dropsy, general | 1 | " hemorrhage | 1 |
| " shot | 1 | " abdomen | 1 | Malformation, general | 1 |
| " run over by Hack | 1 | Dysentery | 2 | Meningitis | 3 |
| " petroleum | 2 | Enteritis | 2 | " cerebro-spinal | 2 |
| " scalded | 2 | Epilepsy | 1 | " tubercular | 5 |
| Abscesses | 1 | Erysipelas | 2 | Necrosis of cranical bone | 1 |
| Abdomen, tumors of | 1 | Fever, congestive | 1 | Old age | 4 |
| Anasarca | 1 | " puerperal, | 3 | Parotitis | 2 |
| Apoplexy | 10 | " intermittent | 2 | Pericarditis | 1 |
| Ascites | 1 | " scarlet | 10 | Peritonitis | 2 |
| Asphyxia | 1 | " malignant | 1 | Peritonitis puerperal | 1 |
| Bowels, gangrene of | 1 | " typhoid | 35 | Peritonaeum inflamm- | |
| Brain, congestion | 3 | " remittent | 3 | tion of | 1 |
| " inflammation | 5 | " complications | 2 | Pharyngitis | 1 |
| " softening | 1 | Hæmatemesis | 1 | Pneumonia | 13 |
| " compression of | 2 | Hemorahage, internal | 2 | " & complications | 2 |
| " disease of | 1 | Heart diseases | 3 | " typhoid | 4 |
| Bronchitis | 4 | " dropsy of | 1 | Pupura haemorrgica | 1 |
| " capillary | 2 | " hypertrophy of | 2 | Pyaemia | 1 |
| " chronic | 3 | " neuralgia of | 1 | Rheumatism | 1 |
| Cancer of liver | 1 | " organic disease of | 1 | Srofula | 2 |
| " stomach | 2 | " valvular disease | 5 | Stomach, chronic inflam- | |
| " uterus | 2 | Hepatitis, | 1 | mation of | 1 |
| " breast | 1 | Hydrocephalus | 1 | Suicide by morphine | 2 |
| Child birth | 1 | " acute | 1 | " drowning | 2 |
| Cholera Infantum | 2 | Inanition | 4 | " arsenic | 1 |
| Consumption | 49 | Intemperance | 1 | Tabes mesenterica | 14 |
| Convulsions | 40 | Icterus | 1 | Teething | 4 |
| " puerperal | 1 | Kidneys, Bright's dis- | | " and complications | 1 |
| Colitis | 2 | ease | 5 | Trichiniasis | 1 |
| Croup | 46 | Larynx contraction of | 1 | Vertebræ, caries of | 1 |
| " diphtheretic | 8 | Larynyitis | 1 | Whooping-cough | 2 |
| Cyanosis | 1 | Liver, disease of | 1 | | ---- |
| Debility general | 7 | " induration | 1 | Total | 422 |

### AGES.

| | | | | | |
|---|---|---|---|---|---|
| Under 1 | 86 | 10 to 20 | 23 | 70 to 80 | 9 |
| 1 to 2 | 54 | 20 to 30 | 44 | 80 to 90 | 4 |
| 2 to 3 | 25 | 30 to 40 | 41 | 90 to 100 | 1 |
| 3 to 4 | 21 | 40 to 50 | 38 | | —— |
| 4 to 5 | 15 | 50 to 60 | 19 | Total, | 422 |
| 5 to 10 | 31 | 60 to 70 | 11 | | |

| | | | | | |
|---|---|---|---|---|---|
| Males, | 231 | Females, | 191 | Total, | 422 |
| Single, | 299 | Married | 123 | Total, | 422 |
| White, | 417 | Colored, | 5 | Total, | 422 |

COMPARISON.

Deaths in Oct., 1870,---422 | Deaths in Oct., 1869, --- 491 | Decrease,---- 69
Deaths in Sept., 1870, ------------ 559 | Decrease,--------------------137

NATIVITY.

| | | | | | |
|---|---|---|---|---|---|
| Austria | 1 | England | 10 | Norway | 10 |
| Bohemia | 7 | France | 1 | Poland | 1 |
| Canada | 6 | Germany | 49 | Russia | 1 |
| Chicago, Native | 58 | Holland | 3 | Switzerland | |
| Chicago, Foreign | 143 | Ireland | 49 | Scotland, | 4 |
| U. S., other parts | 64 | Italy | | Sweden | 8 |
| Denmark | 2 | India | 1 | Unknown | 4 |
| | | | | Total, | 422 |

MORTALITY BY WARDS FOR THE MONTH.

| Wards. | Mortality. | Wards. | Mortality |
|---|---|---|---|
| 1 | 5 | 18 | 33 |
| 2 | 12 | 19 | 7 |
| 3 | 21 | 20 | 11 |
| 4 | 7 | Accidents | 13 |
| 5 | 4 | Bridewell | 1 |
| 6 | 19 | County Hospital | 10 |
| 7 | 15 | Home for Friendless | 1 |
| 8 | 38 | Half Orphan Asylum | 1 |
| 9 | 34 | Marine Hospital | 3 |
| 10 | 22 | Immigrants | 1 |
| 11 | 13 | Scammon Hospital | 1 |
| 12 | 13 | Mercy Hospital | 9 |
| 13 | 8 | Protestant Orphan Asylum | 1 |
| 14 | 9 | St. Joseph Orphan Asylum | 4 |
| 15 | 51 | Suicide | 4 |
| 16 | 20 | | |
| 17 | 32 | Total, | 422 |

When to Give Artificial Food to Infants.—Dr. Brinton (*Trans. Lond. Obstet. Soc.*), advocates giving artificial food to babies, such as corn-flour with milk, when the first teeth come, and the child begins to bite his mother's nipple. When the child's mouth is toothless, its office is to suck the breasts. He orders milk and water until the teeth appear, to infants brought up by hand.

Catheterism of the Larynx.—Dr. Weinlecher, of Vienna, considers catheterism of the larynx, next to tracheotomy, the safest and most rational resort in cases of imminent suffocation from croup and dyptheria.

Receipts from November 25, to December 25, 1870.—Dr. R. N. Isham, City, $6.00; Dr. Theodore Hoffman, City, 3.00; Dr. J. M. Boslaw, Yale, Ill., 3.00; Dr. J. V. Goltsa, Blue Mound, Ill., 3.00; Dr. Radbourg, Diana, Ill., 3.00; Dr. J. Culbertson, Toulon, Ill., 3.00; Dr. A. B. Hanna, Saltello, Tenn., 6.00; Dr. A. L. Marston, Auburn, Wis., 12.00; Dr. J. Guinan, New Lebanon, Wis., 5.00; Dr. W. M. Barbourk, Barrington, Ill., 3.00; Dr. H. L. Butterfield, Waupon, Wis., 6.00; Dr. B. Hawley, Aurora, Ill., 5.00; Dr. L. Carey, Warsen, Ind., 3.00; Dr. E. R. Atwood, Lastorm, Ill., 5.00; Dr. D. Newell, Philiptown, Ill., 3.00; Dr. R. J. Patterson, Batavia, Ill., 3.00; Dr. J. Teffr, Elgin, Ill., 3.00; Dr. J. W. Morrill, New Lisbon, Wis., 3.00; Dr. George R. Bibb, Denver, Col. 6.00; Dr. LaCount, Chilton, Wis., 6.25; Dr. S. D. Mercer, Omaha, Neb., 3.00; Dr. T. P. Sully, City, 12.00; Dr. O. W. Sadler, Byron, Minn., 3.00; Dr. D. W. Stewart, Ottumwa, Iowa, 6.00; Dr. D. C. Roundy, Davenport, Iowa, 6.00; Dr. M. J. Whitman, City, 3.00; Dr. Edward Young, Warren, Ind., 6.00; Dr. W. Fleming, Port Byron, Ill., 6.00; Dr. J. F. Kelsey, Pishtigo, Wis., 3.00; Dr. J. B. LeBland, Brownville, Minn., 3.00; Dr. V. H. Coffman, Omaha, Neb., 4.00; Dr. Curtis, Kansas City, Mo., 3.00.

